# Lysosomal Network Proteins as Potential Novel CSF Biomarkers for Alzheimer’s Disease

**DOI:** 10.1007/s12017-013-8269-3

**Published:** 2013-10-08

**Authors:** Andrea Armstrong, Niklas Mattsson, Hanna Appelqvist, Camilla Janefjord, Linnea Sandin, Lotta Agholme, Bob Olsson, Samuel Svensson, Kaj Blennow, Henrik Zetterberg, Katarina Kågedal

**Affiliations:** 1 Experimental Pathology, Department of Clinical and Experimental Medicine, Faculty of Health Sciences, Linköping University, 581 85 Linköping, Sweden; 2 AlzeCure Foundation, Stockholm, Sweden; 3 Clinical Neurochemistry Laboratory, Department of Neuroscience and Physiology, Sahlgrenska University Hospital, Mölndal, Sweden; 4 San Francisco VA Medical Center, Center for Imaging of Neurodegenerative Diseases (CIND), University of California San Francisco, San Francisco, CA USA; 5 Developmental Biology, Department of Clinical and Experimental Medicine, Linköping University, Linköping, Sweden; 6 UCL Institute of Neurology, Queen Square, London, WC1N 3BG UK

**Keywords:** PICALM, DRAM, TFEB, Cathepsins, Proteasome, hsc70

## Abstract

The success of future intervention strategies for Alzheimer’s disease (AD) will likely rely on the development of treatments starting early in the disease course, before irreversible brain damage occurs. The pre-symptomatic stage of AD occurs at least one decade before the clinical onset, highlighting the need for validated biomarkers that reflect this early period. Reliable biomarkers for AD are also needed in research and clinics for diagnosis, patient stratification, clinical trials, monitoring of disease progression and the development of new treatments. Changes in the lysosomal network, i.e., the endosomal, lysosomal and autophagy systems, are among the first alterations observed in an AD brain. In this study, we performed a targeted search for lysosomal network proteins in human cerebrospinal fluid (CSF). Thirty-four proteins were investigated, and six of them, early endosomal antigen 1 (EEA1), lysosomal-associated membrane proteins 1 and 2 (LAMP-1, LAMP-2), microtubule-associated protein 1 light chain 3 (LC3), Rab3 and Rab7, were significantly increased in the CSF from AD patients compared with neurological controls. These results were confirmed in a validation cohort of CSF samples, and patients with no neurochemical evidence of AD, apart from increased total-tau, were found to have EEA1 levels corresponding to the increased total-tau levels. These findings indicate that increased levels of LAMP-1, LAMP-2, LC3, Rab3 and Rab7 in the CSF might be specific for AD, and increased EEA1 levels may be a sign of general neurodegeneration. These six lysosomal network proteins are potential AD biomarkers and may be used to investigate lysosomal involvement in AD pathogenesis.

## Introduction

Alzheimer’s disease (AD) is a progressive neurodegenerative disease and the most common cause of dementia. The major pathological hallmarks of AD are neurofibrillary tangles composed of hyperphosphorylated tau protein, plaques consisting of aggregated amyloid-β (Aβ) peptides and loss of synapses and neurons in defined areas of the brain (Blennow et al. 2006).

Cerebrospinal fluid (CSF) biomarkers for Aβ and tau are used to diagnose AD in research and increasingly in clinical practice. The presence of increased CSF levels of total-tau (T-tau) and phosphorylated tau (P-tau_181P_) together with lowered levels of Aβ_1–42_ has an 85–95 % accuracy rate for the identification of future AD patients in mild cognitive impairment (MCI) cases (Blennow et al. 2010). However, additional complementary biomarkers are needed to monitor the progression into AD and to stratify possible subpopulations of AD patients. Pre-symptomatic stages occur at least one decade before the clinical onset of AD (Sonnen et al. 2008; Bateman et al. 2012); therefore, there is a clinical need for validated biomarkers that reflect this early stage and shed light on early pathogenic events. The success of future intervention strategies for AD will likely rely on an accurate and early diagnosis with subsequent treatment before the brain is irreversibly damaged.

The “amyloid cascade hypothesis” suggests that an imbalance in the generation and clearance of Aβ causes AD (Hardy and Selkoe 2002; Mawuenyega et al. 2010). However, it is not clear how the production and clearance rates are affected in sporadic AD. Some of the earliest changes in AD are connected to one of the Aβ clearance systems, the lysosomal network, which consists of an interconnected vesicular network of endosomes, lysosomes and autophagosomes (Ihara et al. 2012). The endosomal system is the major sorting compartment that is involved in transport to and from Golgi, internalization and recycling at the plasma membrane and unidirectional transport of proteins marked for lysosomal degradation. Lysosomes contain acid hydrolases that degrade waste materials such as macromolecules, organelles and protein aggregates. The degraded material is recycled in the lysosomes and reused by the cell. Another route for lysosomal delivery is via macroautophagy (hereafter referred to as autophagy). Two other types of autophagy also exist, lysosomal microautophagy and chaperone-mediated autophagy. Autophagy, which is part of normal cell homeostasis, is activated by stress signals such as nutrient starvation, oxidative stress and bacterial infections. During autophagy, long-lived and aggregated proteins or organelles are sequestered in a double-membrane vesicle, called an autophagosome or autophagic vacuole.

Dystrophic neurites in AD show accumulation of large autophagic vacuoles filled with undegraded material, including aggregated Aβ (Nixon et al. 2001). Neuronal endosomal enlargement and the upregulation of genes related to endocytosis and their corresponding proteins are early pathological events in the AD brain (Bronfman et al. 2007; Jiang et al. 2010; Ginsberg et al. 2010). These changes are followed by an increase in lysosomal biogenesis, autophagy impairment and in genes and proteins related to the lysosomal network, suggesting a failure of clearance within the lysosomal system in AD (Boland and Nixon 2006; Nixon et al. 2005; Yu et al. 2005).

The aim of this study was to investigate whether changes in the lysosomal network are mirrored in the CSF of AD patients. A previous study showed that there is an increase in the lysosomal protein cathepsin D in the ventricular CSF from postmortem AD patients (Schwagerl et al. 1995). However, examining biomarkers in postmortem CSF is hazardous due to the rapid leakage of proteins from the autolytic tissue, and another study showed no change in cathepsin B levels in lumbar CSF from living AD patients (Sundelöf et al. 2010). In this paper, we investigated a broad range of lysosomal network proteins (Table 1) and found a significant increase in six of these proteins in the CSF from AD patients when compared with neurological controls (NC).

**Table 1:** Lysosomal network proteins screened, their most frequent cellular compartment association and the primary antibodies used for Western blot. All antibodies were used at a 1:1,000 dilution unless stated otherwise

Target	Product number	Company	Address	Compartment association
Atg4B	Code OSA00033 W/ID tag RB270-270108-WS	Osenses	Keswick, SA, Australia	Autophagosomes
Atg9	Code OSA00042 W/ID tag Rb273-270108-WS	Sigma-Aldrich	St. Louis, MO, USA	Autophagosomes
Atg5	A0731			Autophagosomes
EEA1	E4156			Endosomes
Rab5^a^	R4654			Endosomes
Atg7	ab53255	Abcam	Cambridge, UK	Autophagosomes
IDE	ab133561			Cytosolic
Rab9	ab2810			Endosomes
MHC class II^a^	ab55152			Lysosomes
Atg6^a^	sc11427	Santa Cruz Biotechnology	Santa Cruz, CA, USA	Autophagosomes
hsc70	sc-24			Lysosomes
DRAM	sc-81713			Lysosomes
LIMP-2	sc-55571			Lysosomes
Rab4	sc-312			Endosomes
Rab7	sc-10767			Endosomes
TFEB	sc-4878			Cytosolic/Nuclear
V-ATPase	sc-55544			Lysosomes
PICALM	NBP1-86658	Novus Biologicals	Littleton, CO, USA	Endosomes
LC3	NB600-1384			Autophagosomes
NPC1	NB400-148			Lysosomes
Cathepsin A	AF1049	R&D Systems Inc.	Minneapolis, MN, USA	Lysosomes
Cathepsin B	01-12-030102	Athens Research and Technology	Athens, GA, USA	Lysosomes
Cathepsin D	01-12-030104			Lysosomes
Cathepsin L	01-12-030112			Lysosomes
CD63	556019	BD Biosciences	Franklin Lakes, NJ, USA	Lysosomes
Flotillin 1	610821			Endosomes
LAMP-1	9835-01	Southern Biotech	Birmingham, AL, USA	Lysosomes
LAMP-2	9840-01			Lysosomes
Proteasome 20S	BML-PW8105	Enzo Life Sciences Inc.	Farmingdale, NY, USA	Cytosolic
Rab3	107011	Synaptic Systems GmbH	Goettingen, Germany	Endosomes
SYT7	105172			Lysosomes
Tsg 101	GTX70255	GeneTex Inc.	San Antonio, TX, USA	Endosomes
M6PR	10R-M105a	Fitzgerald	North Acton, MA, USA	Endosomes
ASMase	3687	Cell Signaling Technology	Boston, MA, USA	Lysosomes

^a^Used at 1:500 dilution

## Results

### Increased Levels of the Endosomal Proteins Rab3, Rab7 and EEA1 in AD CSF

**Fig. 1:**
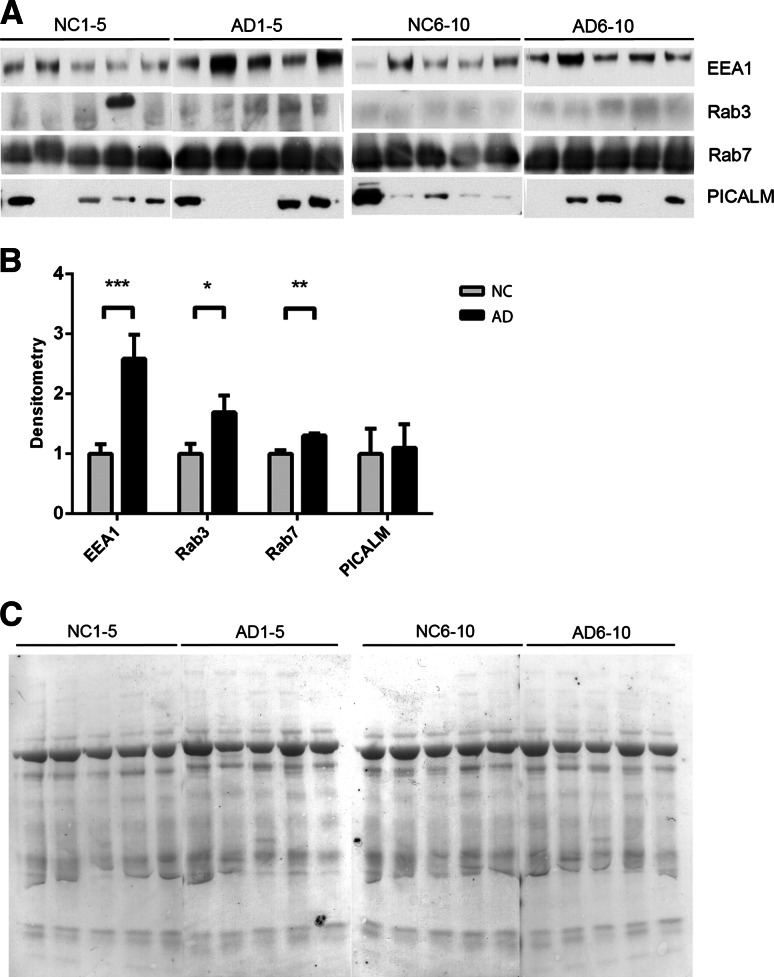
Endosomal proteins EEA1, Rab3 and Rab7 are upregulated in the CSF of AD patients. **a** CSF from ten NC and ten AD patients were analyzed using Western blot for the endosomal proteins EEA1, PICALM, Rab3 and Rab7. **b** Densitometric quantification of the scanned Western blots in **a**. The protein levels are normalized to the means of the respective NC samples. The bars represent the mean ±SD, *p < 0.05, **p < 0.01, ***p < 0.001. **c** Equal sample loading was verified by Ponceau S staining of total protein in each lane on the membranes

We investigated the presence of endosomal proteins in NC and AD CSF (Table 1, CSF set 1) using Western blot analysis. Rab3, Rab7 and early endosome antigen 1 (EEA1) were significantly increased in the AD as compared with the NC CSF samples (Fig. 1a, b). There was up to a 2.6-fold increase in EEA1 levels in AD CSF. For these proteins, there was also a clear difference between the 5 AD and 5 NC samples on each separate blot.

The phosphatidylinositol-binding clathrin assembly protein (PICALM) was present at detectable levels in the CSF, but no significant difference was observed between NC and AD patients. The mannose-6-phosphate receptor (M6PR), Tsg101, Rab4, Rab5, Rab9 and flotillin 1 were not present in the CSF at detectable levels using this method; however, all proteins were detectable in human SH-SY5Y neuroblastoma cell lysates, confirming the ability of the antibodies to recognize the epitopes (data not shown).

### Increased Levels of the Lysosomal Proteins LAMP-1 and LAMP-2 in AD CSF

**Fig. 2:**
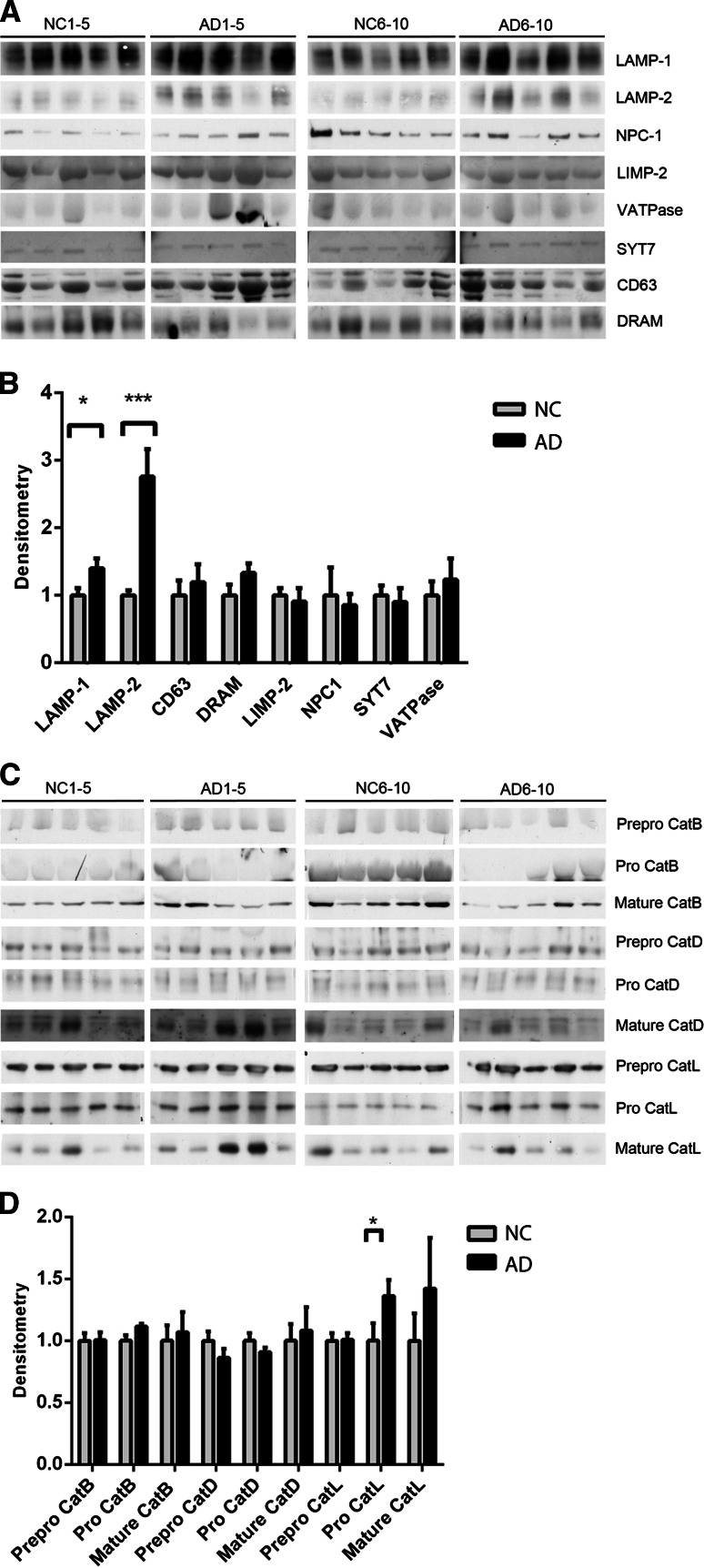
Lysosomal proteins LAMP-1 and LAMP-2 are upregulated in the CSF of AD patients. The CSF from ten NC and ten AD patients were analyzed using Western blot for lysosomal proteins. **a** Western blots of the lysosomal membrane proteins LAMP-1, LAMP-2, CD63, DRAM, LIMP-2, NPC1, SYT7 and V-ATPase. **b** Densitometric quantification of the Western blots in **a**. **c** Western blots of the luminal lysosomal proteases cathepsins B, D and L. **d** Densitometric quantification of the Western blots in **c**. The protein levels are normalized to the means of the respective NC samples. The bars represent the mean ±SD, *p < 0.05, ***p < 0.001

Next, the levels of membrane-bound and luminal lysosomal proteins were investigated. The lysosomal-associated membrane proteins 1 and 2 (LAMP-1 and LAMP-2) were significantly increased in AD patients (Fig. 2a, b) as shown by a 1.4-fold increase in LAMP-1 and a 2.8-fold increase in LAMP-2. For these proteins, there was also a clear difference between the 5 AD and 5 NC samples on each separate blot. The lysosomal membrane proteins CD63, damage-regulated autophagy modulator (DRAM), lysosomal integral membrane protein 2 (LIMP-2), Niemann–Pick type C1 (NPC1), synaptotagmin 7 (SYT7) and vacuolar-type H^+^-ATPase (V-ATPase) were present in the CSF, with a tendency toward increased levels of CD63, DRAM and V-ATPase in AD patients. No detectable levels of acid sphingomyelinase (ASMase) and major histocompatibility complex class II (MHC class II) proteins were found in the CSF; however, the antibodies used detected these proteins in human SH-SY5Y neuroblastoma cell lysates (data not shown).

The luminal lysosomal proteases cathepsins B, D and L were all detectable in CSF. The proform of cathepsin L was significantly upregulated in the CSF from AD patients. No other significant differences were found in the prepro-, pro- and mature forms of the cathepsins between the CSF from NC and AD patients; however, there was a tendency toward an increased expression of the mature forms of the three cathepsins in the CSF of AD patients (Fig. 2a, b). Cathepsin A was not present at detectable levels in CSF. The antibody used detected cathepsin A in human SH-SY5Y neuroblastoma cell lysates (data not shown).

### Increased Levels of the Autophagosomal Protein LC3 in AD CSF

**Fig. 3:**
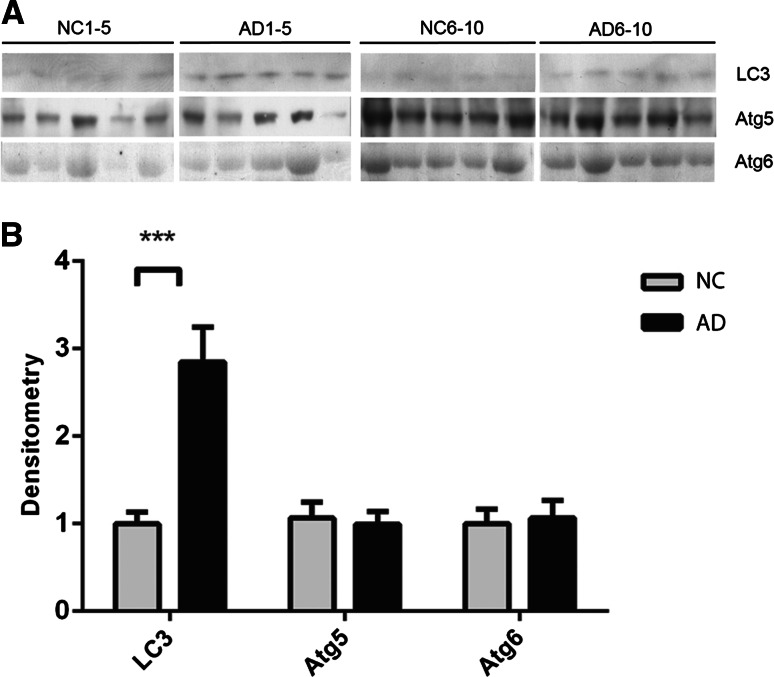
Autophagosomal protein LC3 is upregulated in the CSF of AD patients. **a** CSF from ten NC and ten AD patients were analyzed using Western blot for the autophagosomal proteins LC3, Atg5 and Atg6. **b** Densitometric quantification of the scanned Western blots in **a**. The protein levels are normalized to the means of the respective NC samples. The bars represent mean ±SD, ***p < 0.001

The autophagy-related proteins 4, 5, 6, 7, 8 and 9 (Atg4–9) were analyzed by Western blot. Atg8 (LC3) was significantly upregulated in the CSF of AD patients (Fig. 3a, b) by as much as 2.9-fold. For LC3, there was also a clear difference between the 5 AD and 5 NC samples on each separate blot. Atg5 and Atg6 were present at detectable levels in CSF, but no significant difference was observed between NC and AD samples. Atg4B, Atg7 and Atg9B were not detectable in CSF; however, the antibodies used detected these proteins in human SH-SY5Y neuroblastoma cell lysates (data not shown).

### No Change in Degradation-Associated Proteins hsc70, TFEB and Proteasome 20S in AD CSF

**Fig. 4:**
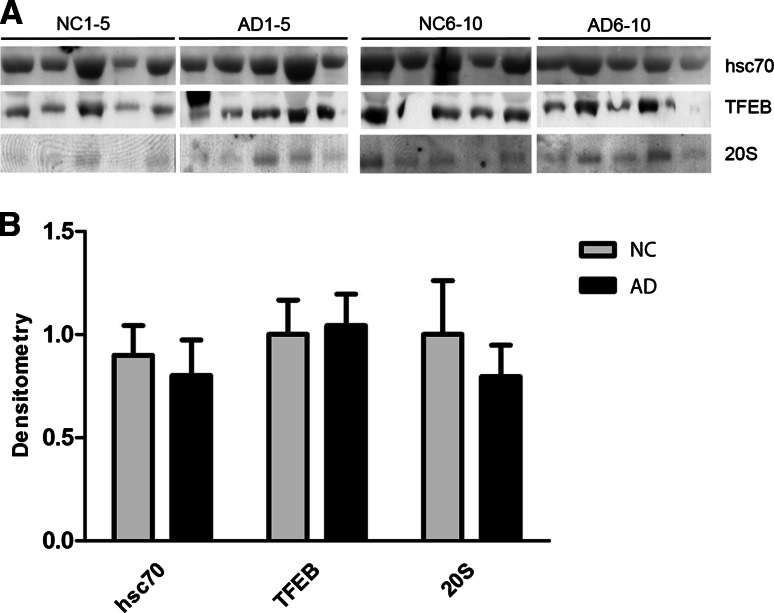
Neither degradation-associated proteins hsc70, TFEB and Proteasome 20S are upregulated in the CSF of AD patients. **a** CSF from ten NC and ten Alzheimer’s disease (AD) patients were analyzed using Western blot for the degradation-associated proteins hsc70, TFEB and Proteasome 20S. **a** Densitometric quantification of the scanned Western blots in **a**. The protein levels are normalized to the means of the respective NC samples. The bars represent the mean ±SD

As described above, we found that the levels of both lysosomal- and autophagy-related proteins were higher in the CSF of AD patients; therefore, we analyzed the master regulator of lysosomal biogenesis and autophagy, the transcription factor EB (TFEB). TFEB was found at detectable levels in the CSF, but no difference was found between NC and AD CSF (Fig. 4a, b). The activation of chaperone-mediated autophagy is often mediated by an increase in LAMP-2 and heat-shock cognate (hsc) 70 proteins. To investigate evidence for chaperone-mediated autophagy, hsc70 levels were analyzed. hsc70 was found at detectable levels in CSF but was not found to be significantly upregulated in the CSF of AD patients (Fig. 4a, b). The other major degradative cellular system is the proteasome; however, the proteasome 20S α2 subunit was analyzed, and no significant change was detected (Fig. 4a, b). The insulin-degrading enzyme (IDE), which efficiently degrades Aβ, was not detectable in the CSF but was detectable in human SH-SY5Y neuroblastoma cell lysates using the same antibody (data not shown).

### Increased Levels of the Six Lysosomal Network Proteins in Another Set of AD CSF Samples

To confirm the findings that EEA1, Rab3, Rab7, LAMP-1, LAMP-2 and LC3 were significantly increased in CSF from AD patients, we tested these proteins in a validation cohort of CSF (Table 2, CSF set 2) from ten NC and ten AD patients. The results confirmed that the same six proteins were significantly upregulated in this second cohort of CSF samples (data not shown).

**Table 2:** Study demographics of AD versus NC (CSF set 1), AD versus NC (CSF set 2) and NC 1–5 from set 1 versus high T-tau (CSF set 3). Data are presented as the mean and (range)

Study group	Age (years)	Sex (M:F)	CSF T-tau (ng/L)	CSF Aβ42 (ng/L)	CSF P-tau_181P_ (ng/L)	Albumin ratio
*CSF set 1*						
AD	68.5	5:5	880	330	123	5.5
*n* = 10	(58–89)		(580–1,680)	(310–390)	(84–177)	(4.2–9.5)
NC	74	6:4	295	780	42	7.8
*n* = 10	(43–82)		(160–390)	(553–1140)	(26–61)	(3.8–10.8)
*CSF set 2*						
AD	73	4:6	844	342	97	7.6
*n* = 10	(50–89)		(467–1,350)	(200–440)	(76–165)	(6.1–10.0)
NC	64	6:4	245	821	39	5.7
*n* = 10	(50–76)		(141–363)	(564–1,150)	(23–60)	(4.0–7.1
*CSF set 3*						
NC 1-5 set 1	72	3:2	271	705	42	6.7
*n* = 5	(61–79)		(170–380)	(553–970)	(26–57)	(3.8–9.2)
High T-tau	65.2	2:3	2541	466	73	n/a
*n* = 5	(26–82)		(487–9,620)	(334–576)	(62–80)	

### LAMP-2 Correlates with the Established Biomarker P-tau_181P_

Next, we examined the correlation of the AD-associated proteins, including EEA1, Rab3, Rab7, LAMP-1, LAMP-2 and LC3, to the previously established AD biomarkers Aβ_1–42_, T-tau and P-tau_181P_. In the AD samples, LAMP-2 was significantly correlated with P-tau_181P_; however, no correlations were detected for Aβ_1–42_ and T-tau (data not shown). No correlations were detected for EEA1, Rab3, Rab7, LAMP-1 and LC3 in the AD samples. In the NC samples, EEA1, Rab3, Rab7 and LC3 were significantly correlated with T-tau, and LC3 was significantly correlated with P-tau_181P_ (data not shown).

### EEA1 Corresponds to Levels of T-tau

**Fig. 5:**
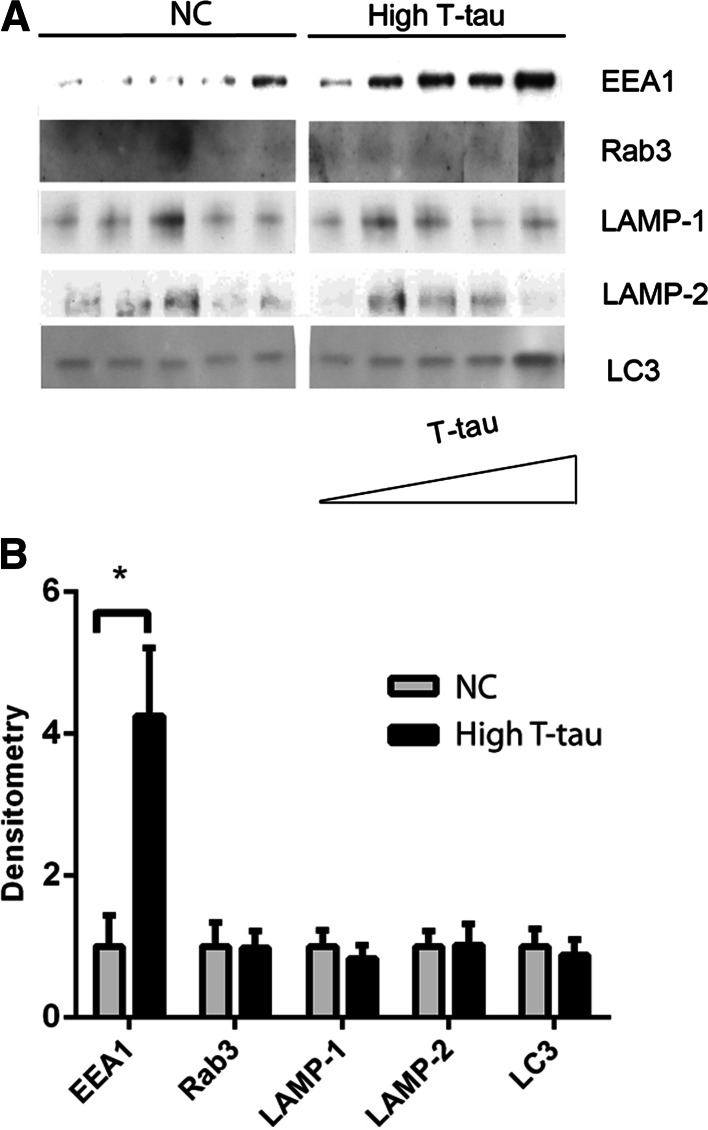
EEA1 levels are enhanced as T-tau levels increase. Five NC samples and five samples with high-T-tau levels (high T-tau) (Table 1, CSF set 3) were analyzed by Western blot. **a** Western blots of EEA1, Rab3, LAMP-1, LAMP-2 and LC3 levels. **b** Densitometric quantification of the scanned Western blots from **a**. The protein levels are normalized to the respective means of the NC samples. The bars represent the mean ±SD, *p < 0.05

To investigate whether EEA1, Rab3, LAMP-1, LAMP-2 and LC3 upregulation were due to general neurodegeneration, the levels of these proteins were analyzed in the CSF of patients that had normal Aβ_1–42_ and P-tau_181P_ levels but elevated T-tau (Table 2, CSF set 3). Due to scarcity of sample, Rab7 could not be measured. EEA1 levels increased as T-tau levels increased (Fig. 5a). The level of EEA1 was also significantly higher in patients with high T-tau (Fig. 5b) when compared with NC samples. There was no detectable difference between NC and high-T-tau samples for Rab3, LAMP-1, LAMP-2 or LC3 (Fig. 5a, b), indicating that these markers have the potential to be specific for AD.

## Discussion

In our current study, we searched for novel AD CSF biomarkers and explored whether changes in the lysosomal network in the AD brain can be monitored using CSF. We identified the endosomal proteins Rab3, Rab7 and EEA1, the lysosomal proteins LAMP-1 and LAMP-2 and the autophagy protein LC3, to be significantly upregulated in the CSF of AD patients. Of thirty-four candidate lysosomal network proteins, we found that only these six proteins were increased, indicating that a specific subset of lysosomal network proteins is overexpressed in the CSF of AD patients. CSF is a natural source for the discovery of new biomarkers because it is relatively easy to obtain with a lumbar puncture. There have been several attempts to analyze the protein content of CSF using proteomics, but the results have been variable. The search is hampered by the fact that more than 70 % of the proteins in CSF are albumin and immunoglobulins and the protein content in CSF tends to vary between individuals (Zougman et al. 2008; Hu et al. 2005). We therefore chose not to perform a high-throughput screen; instead, we used a targeted Western blot analysis of proteins associated with the lysosomal network.

 In neurons, endocytosis plays a critical role in synaptic transmission and plasticity, neuronal development and homeostasis (Delcroix et al. 2003) (Bronfman et al. 2007). Endosomal changes are among the earliest pathologies observed in the AD brain. Increased PICALM transcription levels have been reported in AD (Baig et al. 2010), EEA1 transcription levels have been found to be increased in MCI and AD (Jiang et al. 2010), and Rab4, Rab5 and Rab7 are upregulated in MCI and AD (Jiang et al. 2010; Ginsberg et al. 2010) and abnormally recruited to endosomes in AD (Cataldo et al. 1997, 2000; Yang et al. 2008). In contrast, Rab3a was found to be decreased in specific degenerative areas of AD brains (Sze et al. 2000; Davidsson et al. 2001; Blennow et al. 1996). Similar to previous findings, we determined that EEA1 and Rab7 were significantly increased in AD CSF. However, we also observed an increased level of Rab3 in AD CSF. This could be due to compensatory mechanisms that precede the loss of synapses in the AD brain or that brain areas described to have decreased levels of Rab3 are relatively small, and a change might not be mirrored in the CSF. Flotillin 1, Rab4 and Rab5 were previously described to be linked to AD, but all these proteins were below the detection limit.

The lysosomal system is altered in AD. The lysosomal membrane protein LAMP-1 was previously shown to be upregulated at both the mRNA and protein level in the AD brain (Barrachina et al. 2006). In our current screen, LAMP-1 was found to be increased in the AD CSF; in addition, LAMP-2 was also increased in AD CSF. LAMP-2 has been reported to be increased in several lysosomal storage disorders as a general response to decreased lysosomal clearance (Hua et al. 1998), but has not previously been indicated as upregulated in AD. The prior finding that the lysosomal membrane protein NPC1 is elevated in AD brains (Kågedal et al. 2010) was not reflected in the AD CSF samples used in this study. Cathepsins B and D have been shown to be increased in AD brains (Cataldo et al. 1997). Cathepsin B was detected in AD CSF, but no increase in expression level was found (Sundelöf et al. 2010), and cathepsin D levels exhibited a fourfold increase in ventricular CSF collected from postmortem AD patients (Schwagerl et al. 1995). We could not detect any significant expression changes in cathepsins B, D or L, except that the proform of cathepsin L was higher in AD CSF. However, all mature forms of the cathepsins were slightly elevated in AD CSF. Although they were all detectable in CSF, no alterations of the lysosomal membrane proteins V-ATPase, CD63, DRAM, LIMP-2, SYT7 or M6PR could be found.

Different types of autophagic vacuoles have been identified in AD biopsies (Terry et al. 1964; Boland and Nixon 2006). Autophagy is upregulated in AD brains, reflecting either the induction of autophagy or a reduction in the turnover of autophagosomes (Nixon et al. 2005; Lipinski et al. 2010). The different steps in the autophagy process are tightly regulated by a family of Atg proteins (Klionsky et al. 2003). We investigated the levels of Atg4, 5, 6, 7, 8 and 9 and found that Atg8 (LC3) was significantly upregulated in AD CSF. LC3 has been linked to AD tauopathies, but has not been previously found in the CSF.

We also investigated the levels of proteins involved in proteolytic degradation and Aβ clearance, including TFEB, IDE, the proteasome 20S subunit and hsc70. Previously, the proteasome 20S was found to be increased in the AD brain, and the proteasome also was reported to be altered in neuroinflammatory disease (Upadhya and Hegde 2007; Ciechanover and Brundin 2003). IDE is an Aβ-degrading protease that was reported to be decreased in the brain of MCI and AD patients (Perez et al. 2000). In our study, all of the above proteins, apart from IDE, were detected in the CSF; however, there were no significant differences between AD and NC samples.

A correlation analysis of our six lysosomal network proteins and the established AD biomarkers, Aβ, T-tau and P-tau_181P_, in AD CSF showed that LAMP-2 correlates significantly in a positive fashion with P-tau_181P_. There are indications that CSF P-tau_181P_ can be used to differentiate AD from other dementias (Hampel et al. 2004), and it will be interesting to investigate whether LAMP-2 can be similarly used as a more specific marker for AD. None of the six proteins correlated with the Aβ_1–42_ level, most likely because all AD samples had similar Aβ_1–42_ levels (310–390 ng/L), making the range for correlation analysis narrow. While many of the proteins in the NC samples (EEA1, Rab3, Rab7 and LC3) correlated with T-tau, it is interesting that none of the proteins in the AD samples did. One possible interpretation is that tau and these proteins are normally metabolized through coordinated pathways, which are disturbed in AD. The loss of correlation with T-tau suggests that mechanisms other than pure neurodegeneration may affect the CSF level of these proteins in AD and may be a disease marker in itself.

To investigate the AD specificity of the six lysosomal network proteins, we analyzed the levels of the proteins in patients with increased T-tau values but normal Aβ_1–42_ and P-tau_181P_ values (i.e., patients suffering axonal damage likely not caused by AD). The only protein that correlated with T-tau was EEA1, which might imply that EEA1 levels in the CSF reflect general neurodegeneration, and could potentially be used to monitor the onset of the disease. The endosomal system is changed early in AD pathogenesis, but not in other neurodegenerative disorders, such as encephalitis, Lewy body dementia and Huntington’s disease (Cataldo et al. 2000). Therefore, there is a possibility that Rab3 and Rab7 are specific to AD and change early in disease progression. To date, no biomarker has been identified that monitors the progression of AD. EEA1, LAMP-1, LAMP-2 and LC3 have the potential to be used to monitor the disease progression because lysosomal network changes in AD are progressive with slow changes, leading to a buildup of incompletely digested substrates within neuronal processes.

One limitation of our study is the limited clinical information available on the included subjects. All sought medical advice because of minor cognitive, psychiatric or neurological symptoms and were grouped into AD or NC on the basis of CSF biomarker results that are 80–95 % accurate for AD according to cut-points established within our laboratory on external cohorts (Zetterberg et al. 2003, Hansson et al. 2006). Recent data suggest that these biomarker patterns may in fact be more accurate than a clinical diagnosis to identify or exclude AD neuropathology (Andreasson et al. 2013). However, it will be important to validate the findings in clinically diagnosed patients in future studies.

All antibodies used were confirmed to recognize their epitope, so not detecting some of the proteins in the CSF could be either that they were not present or due to the sensitivity of our method, which might have been improved if the CSF had been concentrated, or from changes in the protein to freeze–thawing. Also, some proteins that were previously described as altered in the AD brain were not changed in CSF. The lack of change might be due to the small sample sizes used in previous studies or the relatively small sample size in our study, and also that not all protein changes in the brain tissue are mirrored in the CSF. In contrast to brain material, the CSF can be sampled from live patients, making detectable biomarkers highly valuable.

AD is a neurodegenerative disease with a severe loss of neurons. Early diagnostics are crucial to start treatment before the brain is irreversibly damaged. Today, an accurate diagnosis of AD is not possible until postmortem; therefore, reliable biomarkers for AD are highly needed in research and clinics for diagnosis, patient stratification, clinical trials, monitoring of disease progression and the development of new treatment strategies. Therefore, it is important to explore biomarkers that identify proteins that most likely reflect early AD pathology. We are the first to report that six specific lysosomal network proteins are overexpressed in the CSF of AD patients. The Rab3, Rab7, EEA1, LAMP-1, LAMP-2 and LC3 proteins may be valuable as tools for the investigation into the role of endosomal, lysosomal and autophagy pathways in vivo in human AD patients.

## Materials and Methods

### Study Population

The initial study was performed on twenty de-identified, archived CSF samples (Table 2, CSF set 1), and the results were confirmed in an unrelated set of twenty CSF samples (Table 2, CSF set 2). In addition, five high-T-tau samples were tested (Table 2, CSF set 3). All samples were from the Clinical Neurochemistry Laboratory, Sahlgrenska University Hospital/Mölndal, Sweden. The samples were collected from patients who sought medical advice because of minor cognitive or neurological symptoms. Following clinical routine, the CSF AD biomarkers P-tau_181P_, T-tau and Aβ_1–42_ were analyzed in these patients. Based on their neurochemical profile, the samples were designated as AD (*n* = 10), NC (*n* = 10) or high T-tau (*n* = 5). AD samples were designated according to CSF biomarker levels using cutoffs that have 95 % sensitivity and >80 % specificity for AD, including T-tau >350 ng/L, Aβ_1–42_ <530 ng/L and P-tau_181P_ >60 ng/L (Hansson et al. 2006), and the patients were abnormal in all of these parameters. The CSF samples that were largely negative for changes in these parameters were designated NC. Among the NC subjects, two patients had slightly elevated T-tau, and one patient had P-tau_181P_ at the reference limit. The remaining NC subjects were neurochemically normal. The samples designated as high T-tau had elevated T-tau values, but the Aβ_1–42_ and P-tau_181P_ values were in the same range as the NCs. The CSF samples used are found in Table 2.

### CSF AD Biomarkers

CSF was collected in polypropylene tubes, centrifuged and frozen, later thawed, aliquoted and restored at −80 °C pending analysis. CSF levels of the core AD biomarkers, including Aβ_1–42_, T-tau and P-tau_181P_ phosphorylated at threonine_181_, were determined using INNOTEST^®^ ELISA kits as previously described (Blennow et al. 1995; Vandermeeren et al. 1993; Vanderstichele et al. 2000; Vanmechelen et al. 2000) and according to the instructions from the manufacturer (Innogenetics, Ghent, Belgium). Albumin levels were measured by immunonephelometry on a Beckman Image Immunochemistry system (Beckman Instruments, Beckman Coulter, Brea, CA, USA). The albumin ratio was calculated as CSF albumin/serum albumin and was used as a measure of blood–brain barrier function. All analyses were performed by certified laboratory technicians blinded to the specifics of the clinical data. Samples were analyzed in clinical routine in several analytical runs, but an elaborate system of internal and external quality control samples were used to assure low variability for each of the measurements. The detailed methods were approved by the Swedish Board for Accreditation and Conformity Assessment (SWEDAC).

### Western Blotting

Twenty microliters of CSF was mixed with loading buffer (0.1 M Tris, pH 6.8, 6 % glycerol, 4 % SDS, 0.2 % bromophenol blue, 1.6 % β-mercaptoethanol, 50 mM DTT), and the samples were heated to 95 °C. Proteins were separated by SDS-PAGE (200 V, 90 mA/gel, 1 h) and blotted onto a nitrocellulose membrane using the Invitrogen iBlot^®^ Dry Blotting System (Invitrogen, Paisley, UK). The membrane was blocked in 5 % dry milk or 5 % BSA in TBS-Tween and probed with a primary antibody (diluted in 5 % dry milk or 5 % BSA in TBS-Tween) overnight at 4 °C. The membranes were washed and probed with horseradish peroxidase-conjugated rabbit anti-goat antibodies, goat anti-rabbit or goat anti-mouse antibodies (P0449, P0448 and P0447, Dako, Glostrup, Denmark) for 1 h at room temperature. After washing, immunodetection of the bound antibodies was performed using Amersham™ ECL™ detection systems (GE Health Care, Pittsburgh, PA, USA) and exposure to photographic film. The primary antibodies used (shown in Table 1) were confirmed to recognize their epitope via Western blot analysis of cell lysates. Equal sample loading was verified by Ponceau S (Sigma-Aldrich, St. Louis, MO, USA) staining of total protein in each lane on the membranes (representative blot in Fig. 1c). The films were scanned and the immunoblots were quantified using the Image J program (available at http://rsbweb.nih.gov/ij/). The relative amount of protein corresponding to an immunoreactive band was calculated as a product of average optical density of the area of the band. The samples were ran on two gels on the same day by the same person and developed together on the same film for the same amount of time. Each gel was loaded with 5 NC and 5 AD samples, and any difference in blot intensity, due to development differences between the paired gels, was removed by subtracting the blot background for each sample separately.

### Statistical Analysis

The mean value and standard deviation (SD) were calculated for all data. The nonparametric Mann–Whitney *U* test was used to test for significant differences between groups. Correlation analysis to measure dependence between two quantities was performed using the Spearman’s rank correlation coefficient. Statistical significance was defined for *p*-values of less than 0.05 (*), 0.01 (**) and 0.001 (***). All statistical analyses were performed using GraphPad Prism version 5.00 for Windows (GraphPad Software, San Diego, CA USA). There was a clear difference between AD and NC samples on each separate gel, and we could therefore perform statistical testing on all 20 samples together.
